# Supplementary material for: GATA6 regulates WNT and BMP programs to pattern precardiac mesoderm during the earliest stages of human cardiogenesis
Source: eLife. 2025 Mar 13;13:RP100797. doi: 10.7554/eLife.100797 (PMC11906159; doi:10.7554/eLife.100797)
Supplement: Figure 1—figure supplement 1—source data 1. [file elife-100797-fig1-figsupp1-data1.pdf]

Figure 1-figure supplement 1 – Source Data 1

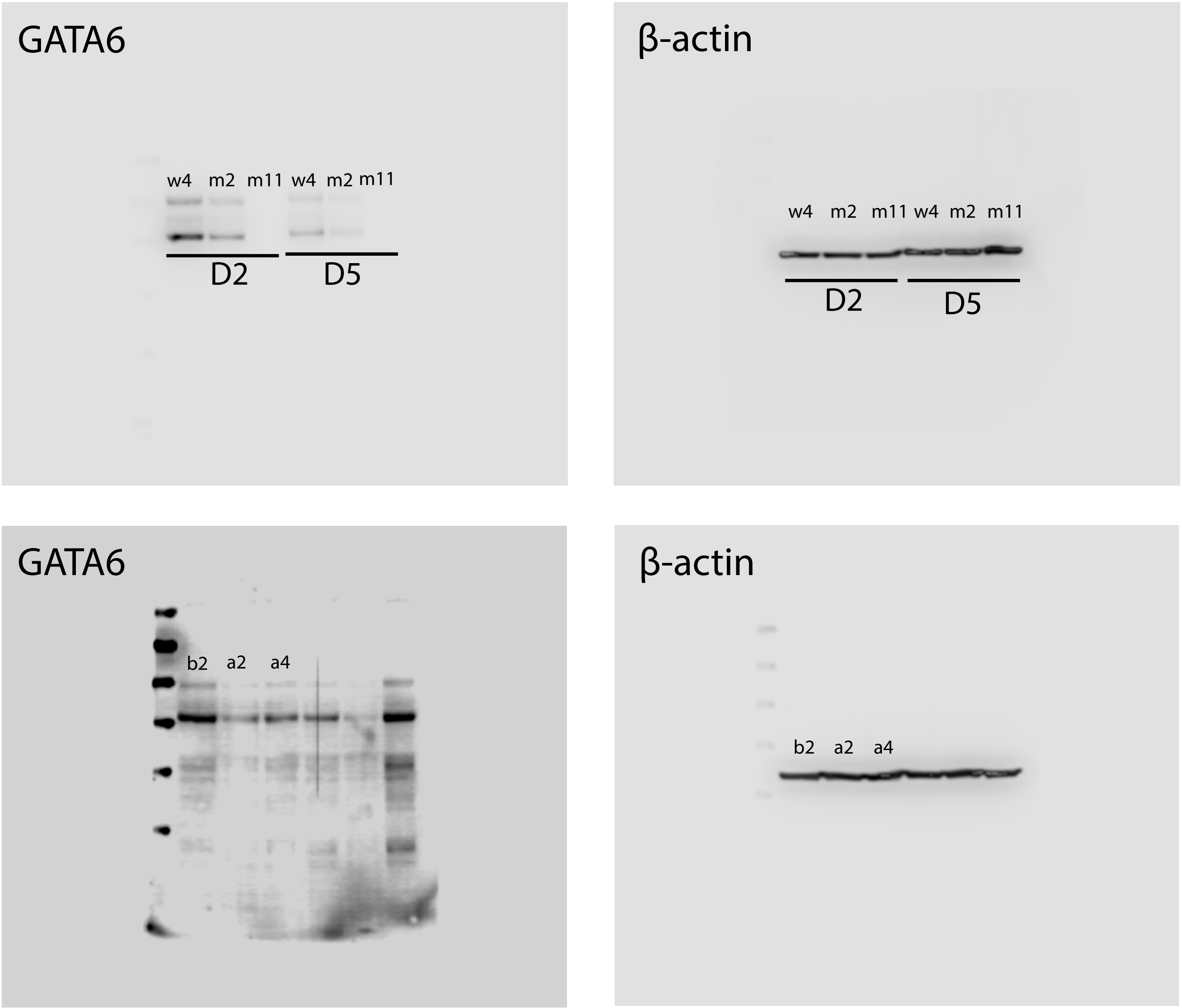

Figure 1-figure supplement 1 – Source Data 1. Original western blot images corresponding to Figure 1-figure supplement 1C (top two images) and Figure 1-figure supplement 1K (bottom two images). Labels indicate the antibody used (top left corners), hPSC clonal line used, and/or day of cardiac differentiation.
