# Supplementary figures and images for: GATA6 regulates WNT and BMP programs to pattern precardiac mesoderm during the earliest stages of human cardiogenesis
Source: eLife. 2025 Mar 13;13:RP100797. doi: 10.7554/eLife.100797 (PMC11906159; doi:10.7554/eLife.100797)

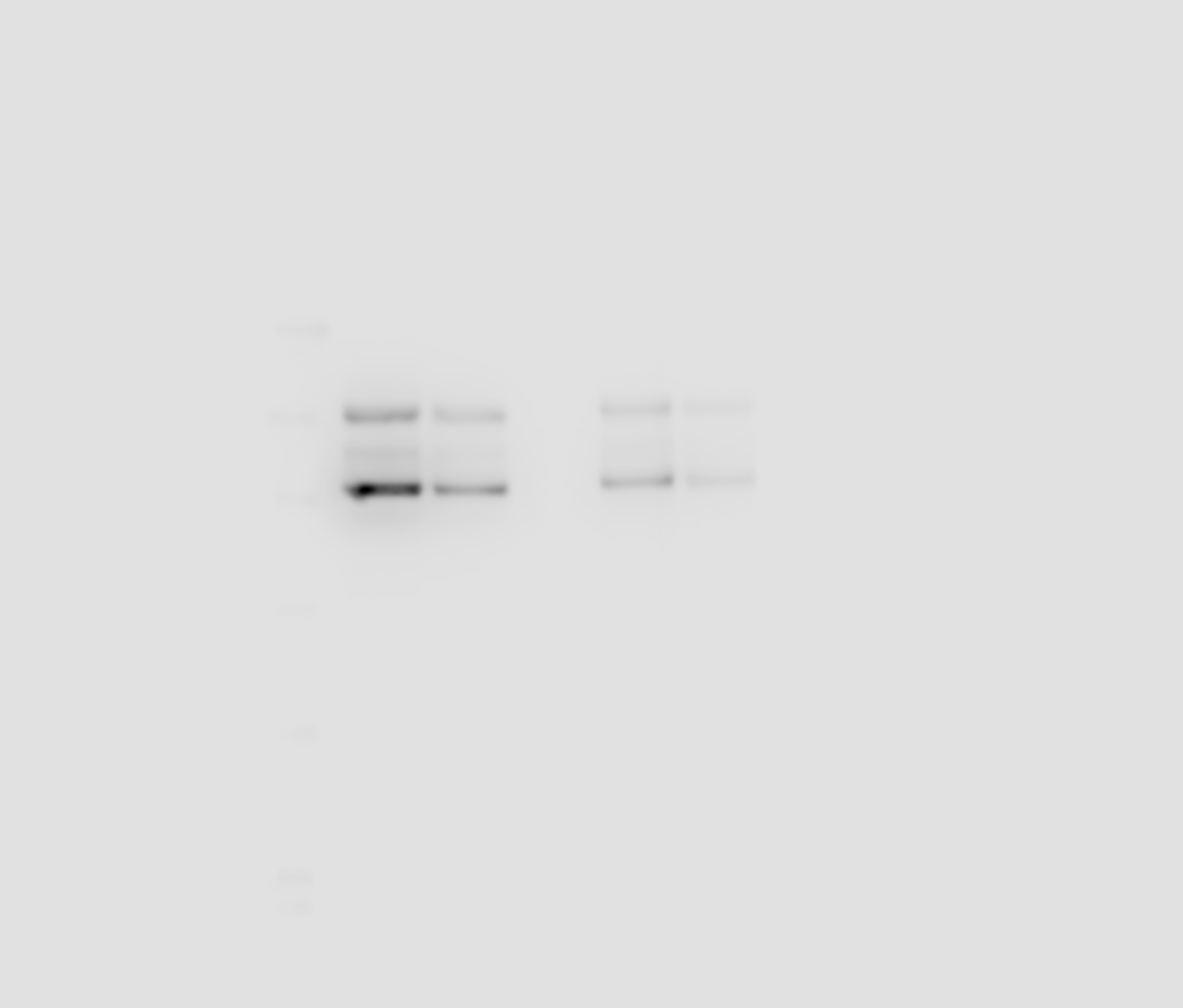

Supplement: Figure 1—figure supplement 1—source data 3. [file elife-100797-fig1-figsupp1-data3.zip › Figure 1-figure supplement 1 - Source Data 2/wt het mut d2 d5 G6.tif]

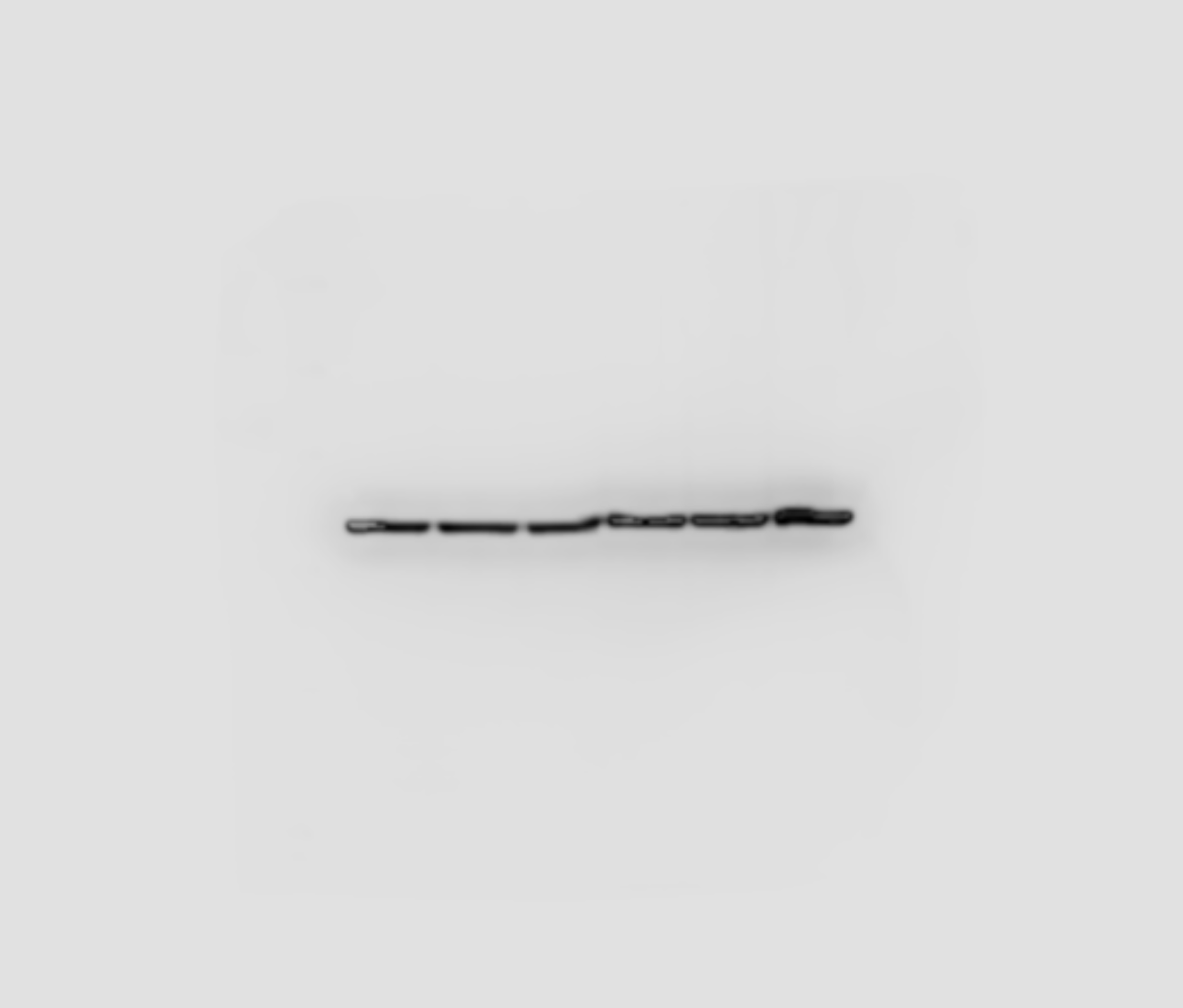

Supplement: Figure 1—figure supplement 1—source data 3. [file elife-100797-fig1-figsupp1-data3.zip › Figure 1-figure supplement 1 - Source Data 2/wt het mut d2 d5 bactin.tif]

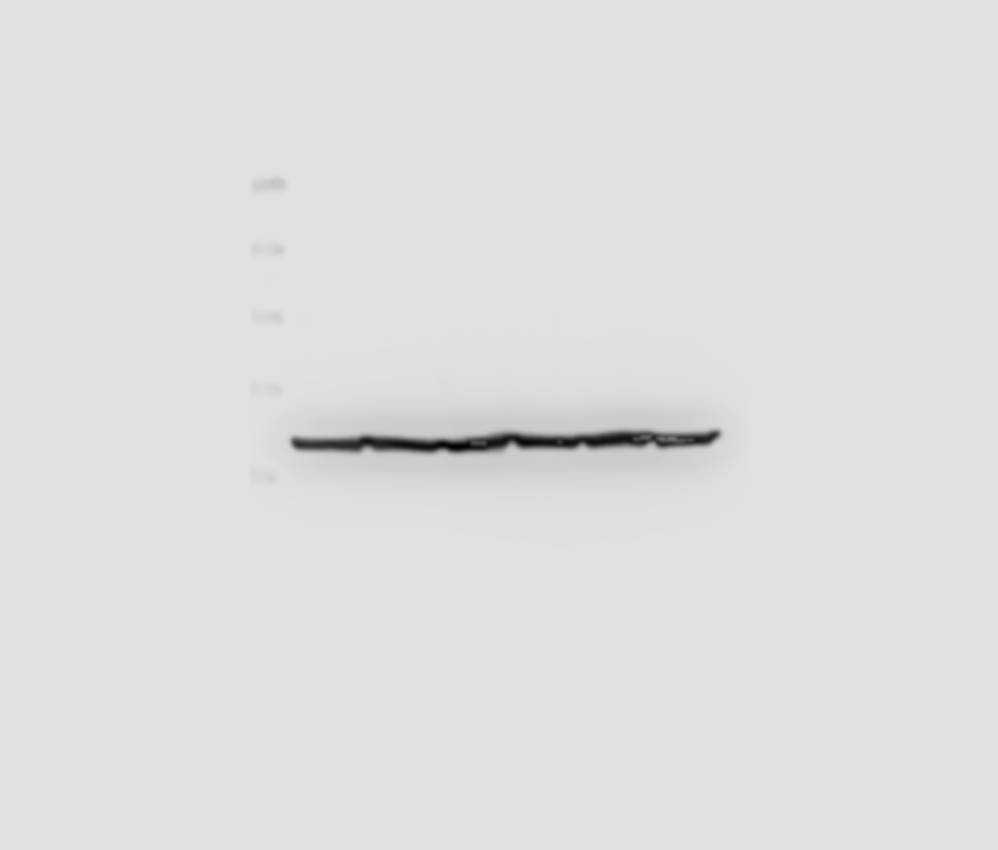

Supplement: Figure 1—figure supplement 1—source data 3. [file elife-100797-fig1-figsupp1-data3.zip › Figure 1-figure supplement 1 - Source Data 2/G6 iPSb-actin.png]

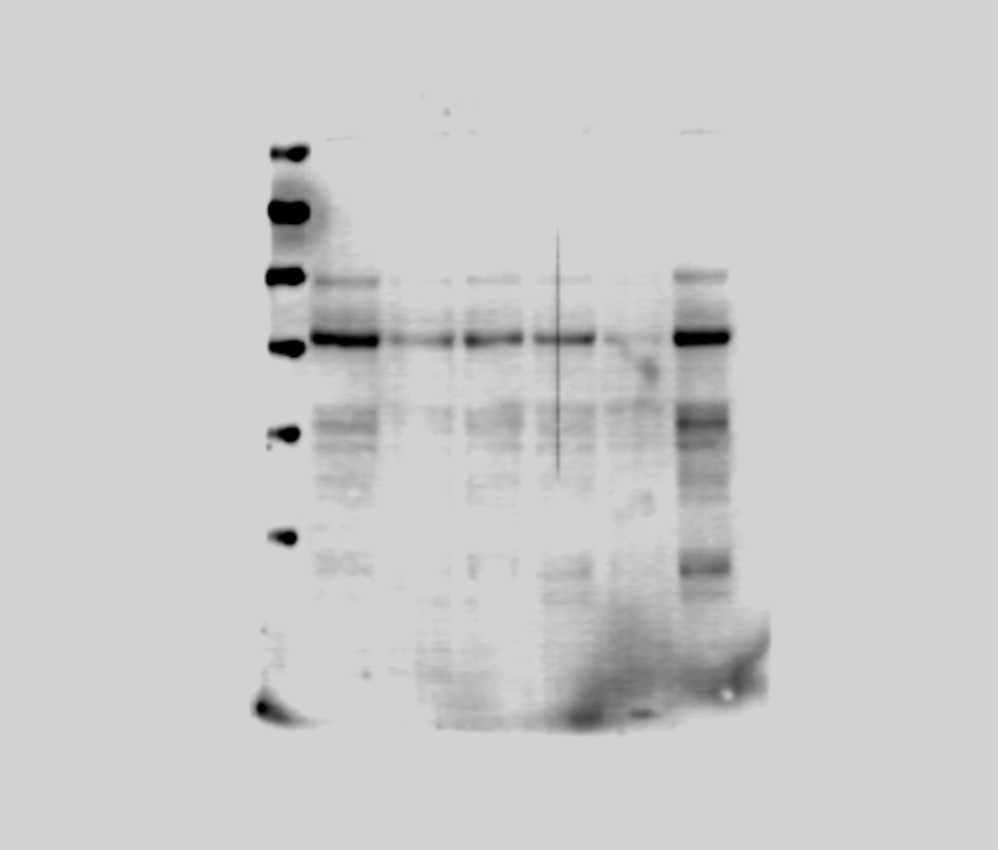

Supplement: Figure 1—figure supplement 1—source data 3. [file elife-100797-fig1-figsupp1-data3.zip › Figure 1-figure supplement 1 - Source Data 2/G6 iPS gata6 b2 a2 a4.png]

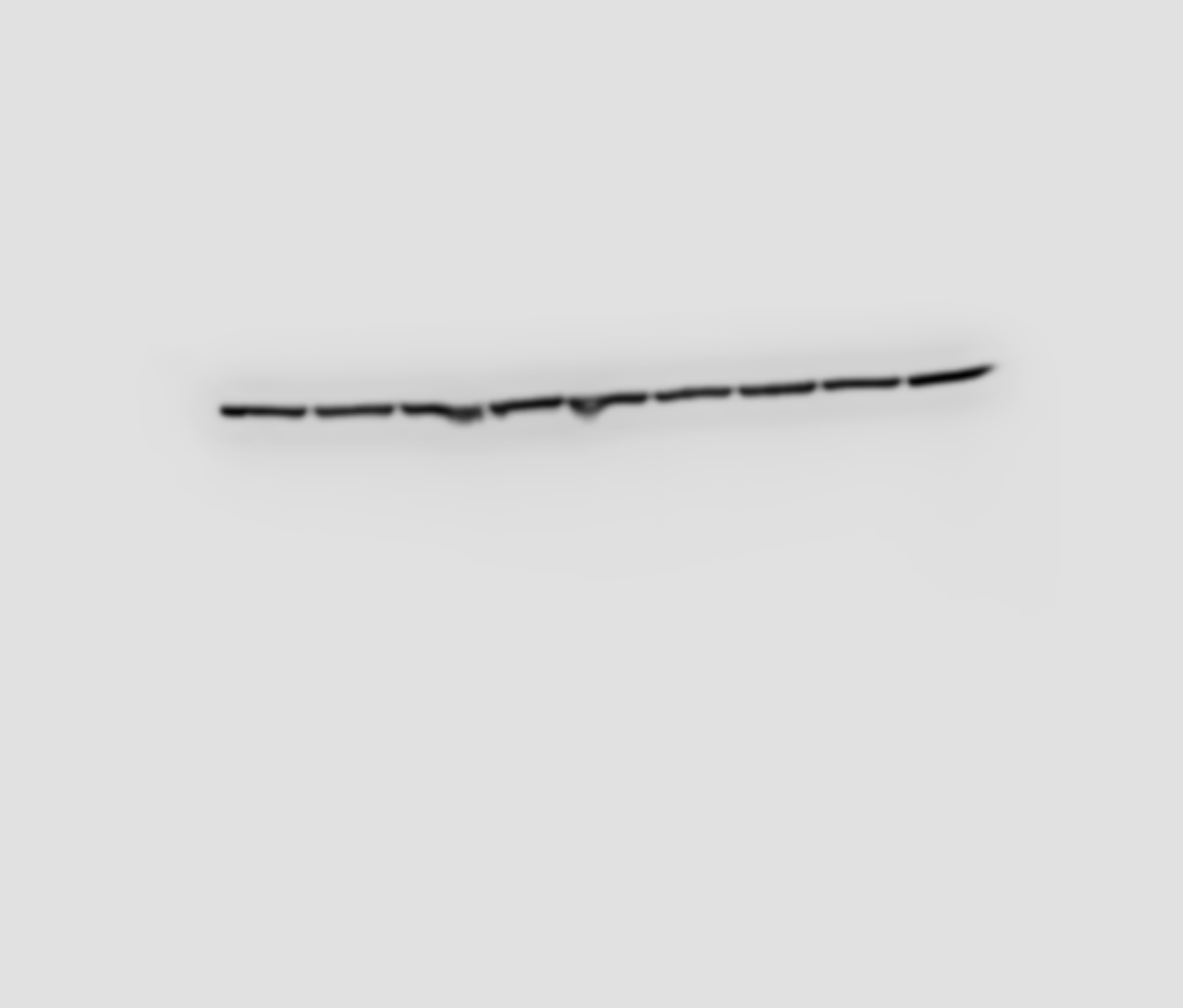

Supplement: Figure 3—source data 2. [file elife-100797-fig3-data2.zip › Figure 3 - Source Data 2/g6 wt het ko bactin.png]

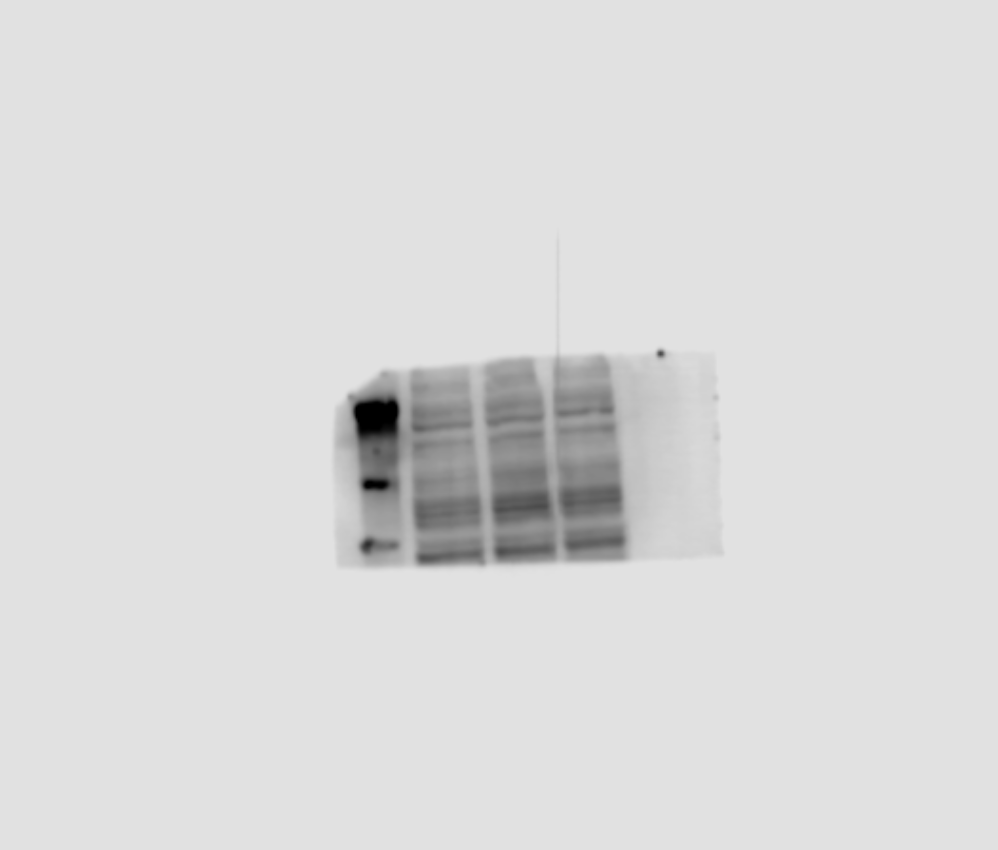

Supplement: Figure 3—source data 2. [file elife-100797-fig3-data2.zip › Figure 3 - Source Data 2/pSMAD2-3.png]

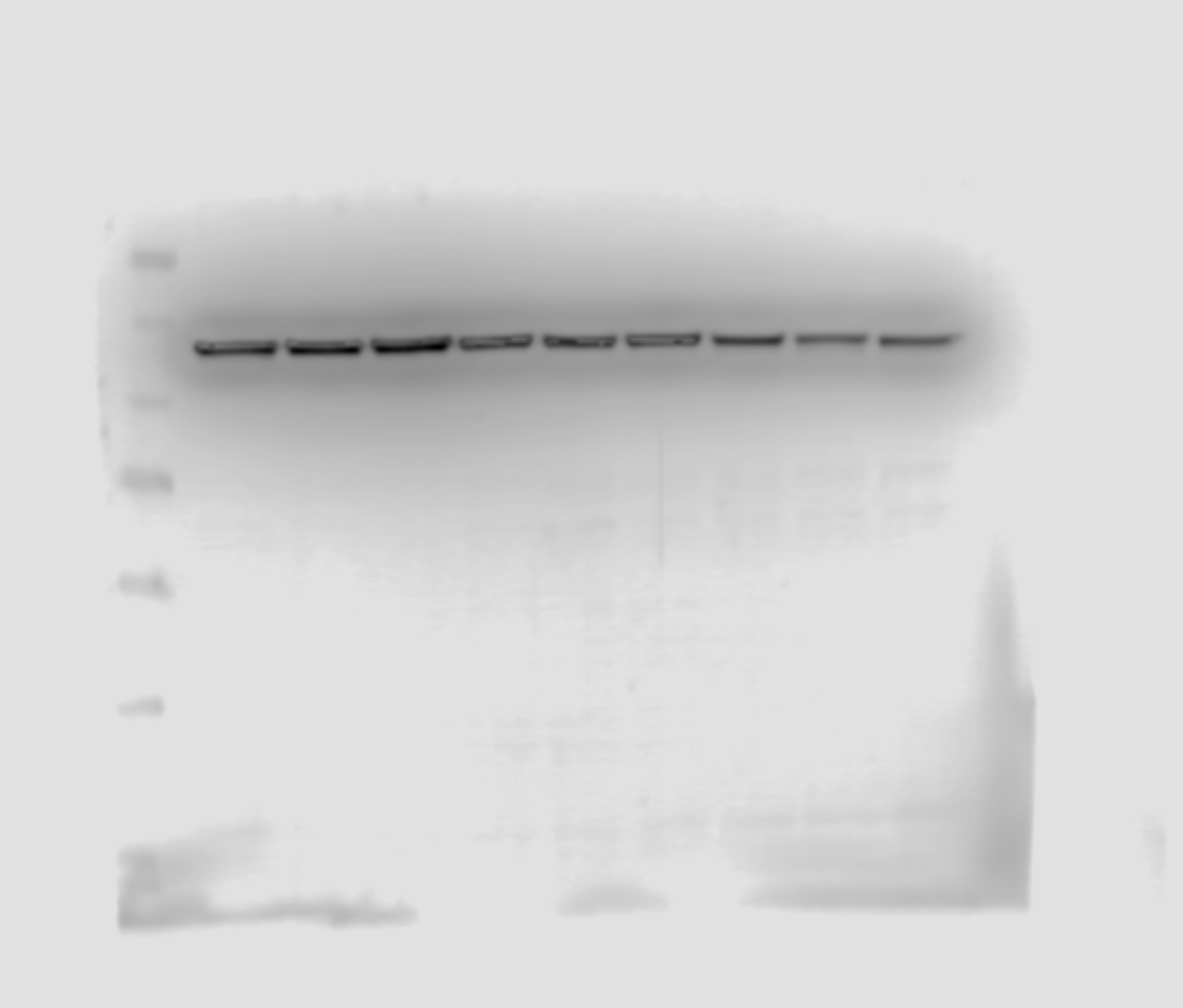

Supplement: Figure 3—source data 2. [file elife-100797-fig3-data2.zip › Figure 3 - Source Data 2/g6 wt het ko nonphos bcat.png]

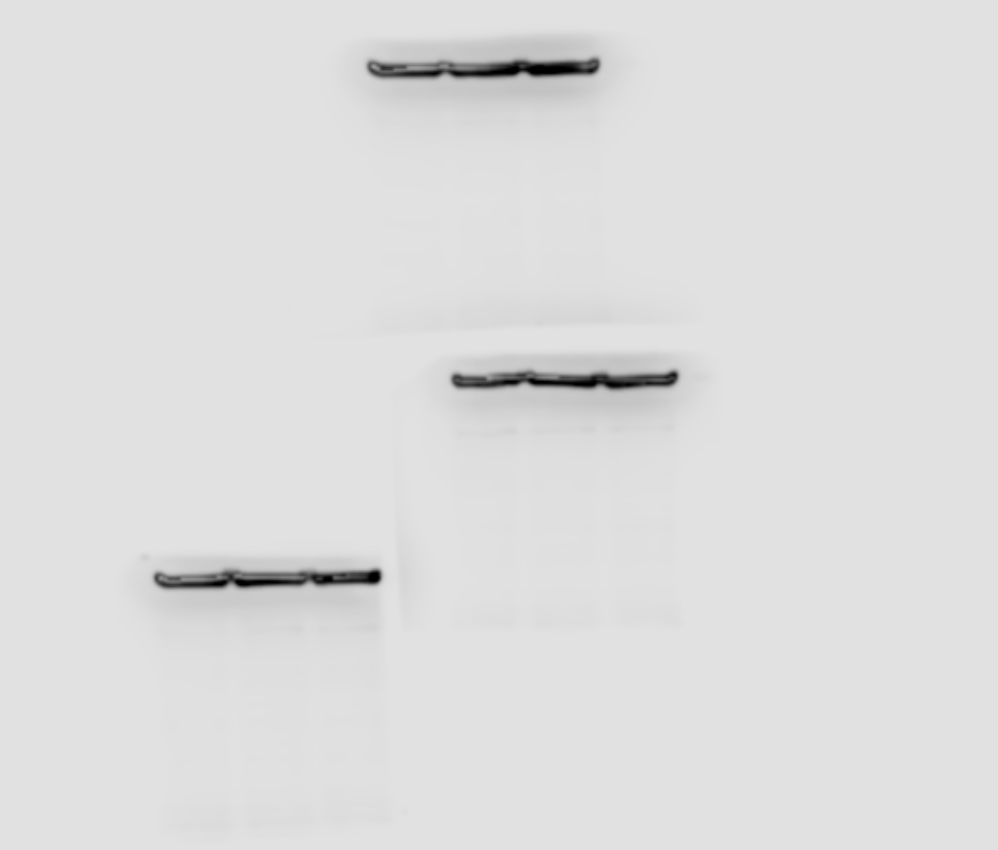

Supplement: Figure 3—source data 2. [file elife-100797-fig3-data2.zip › Figure 3 - Source Data 2/SMAD- b-actin blots.png]

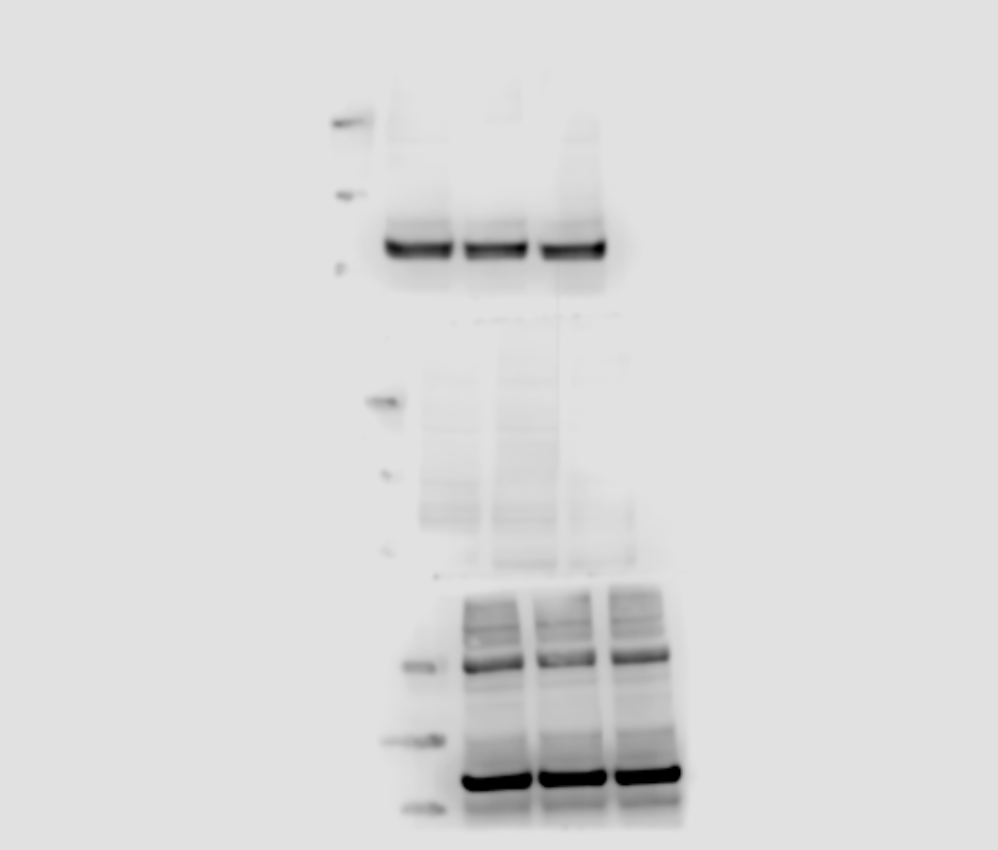

Supplement: Figure 3—source data 2. [file elife-100797-fig3-data2.zip › Figure 3 - Source Data 2/pSMAD 1-5-9 top.png]

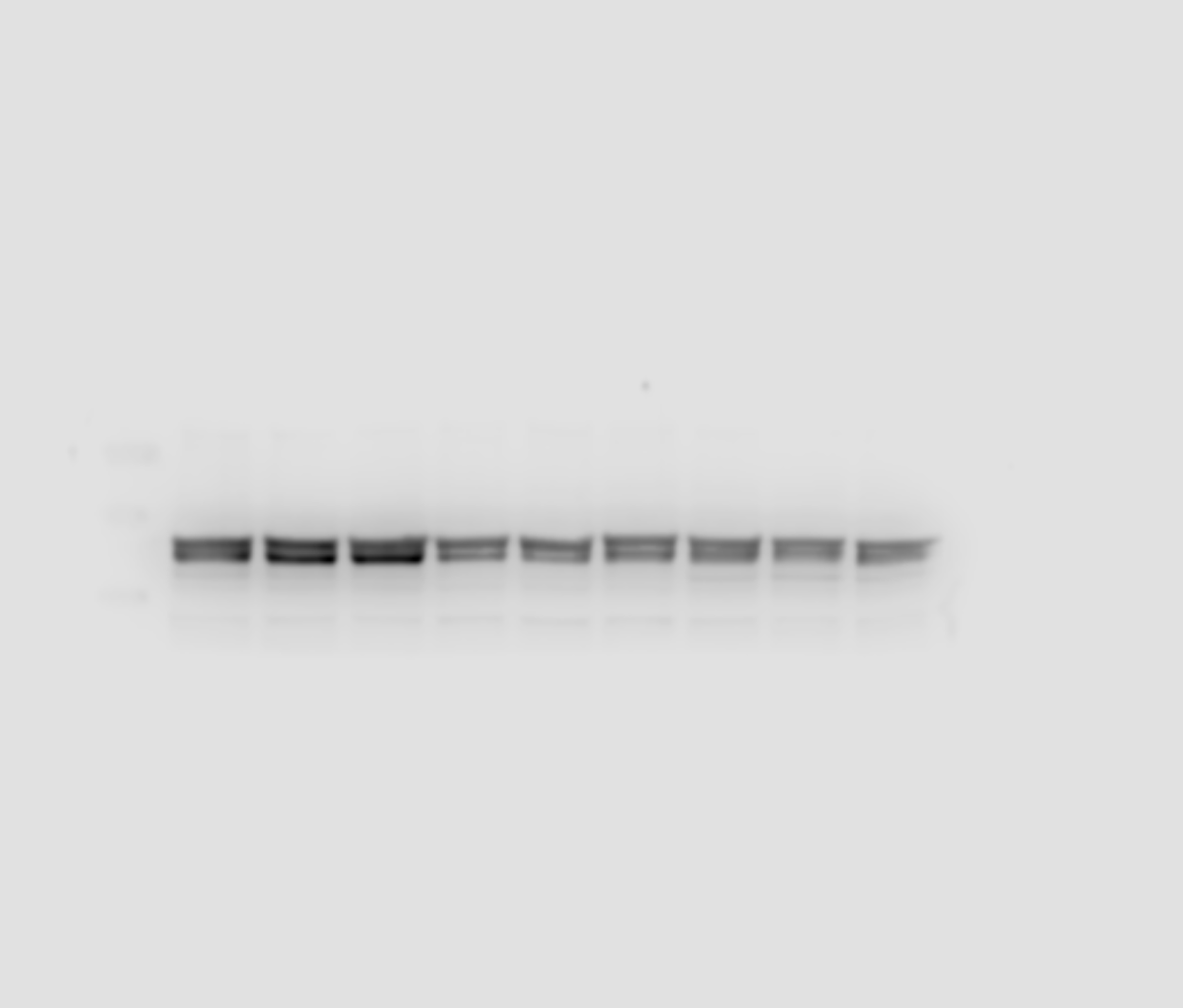

Supplement: Figure 3—source data 2. [file elife-100797-fig3-data2.zip › Figure 3 - Source Data 2/g6 wt het ko total beta catenin.png]

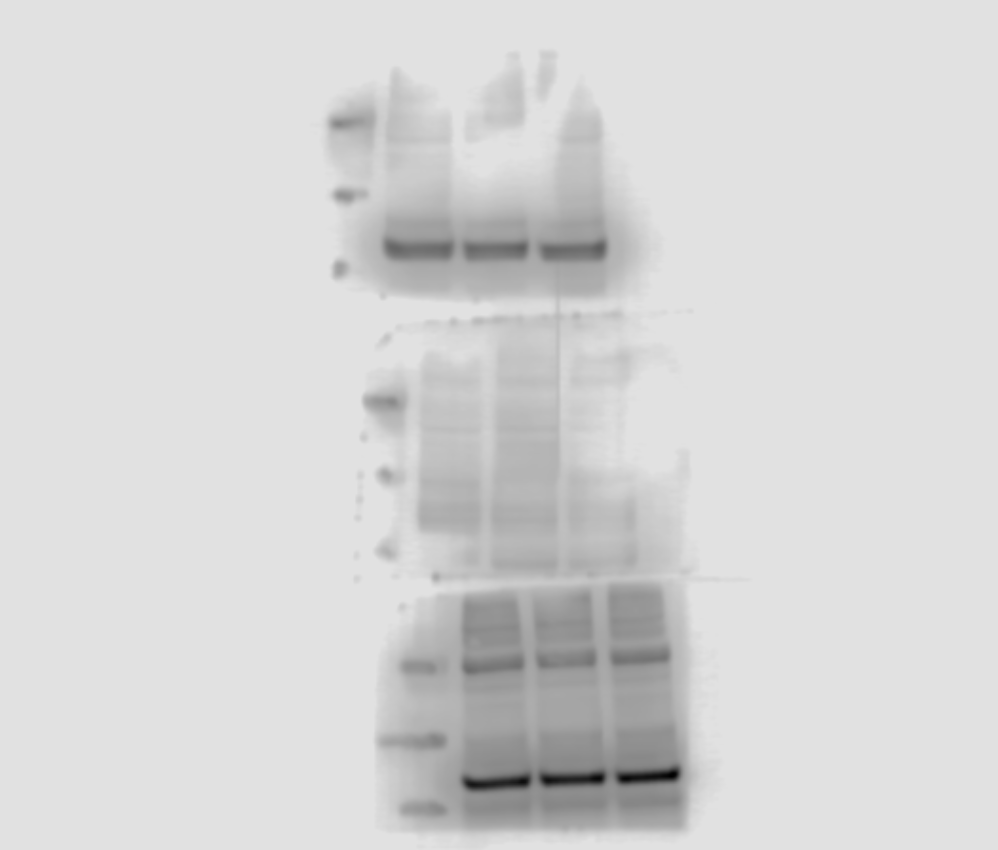

Supplement: Figure 3—source data 2. [file elife-100797-fig3-data2.zip › Figure 3 - Source Data 2/total SMAD2-3 bottom.png]

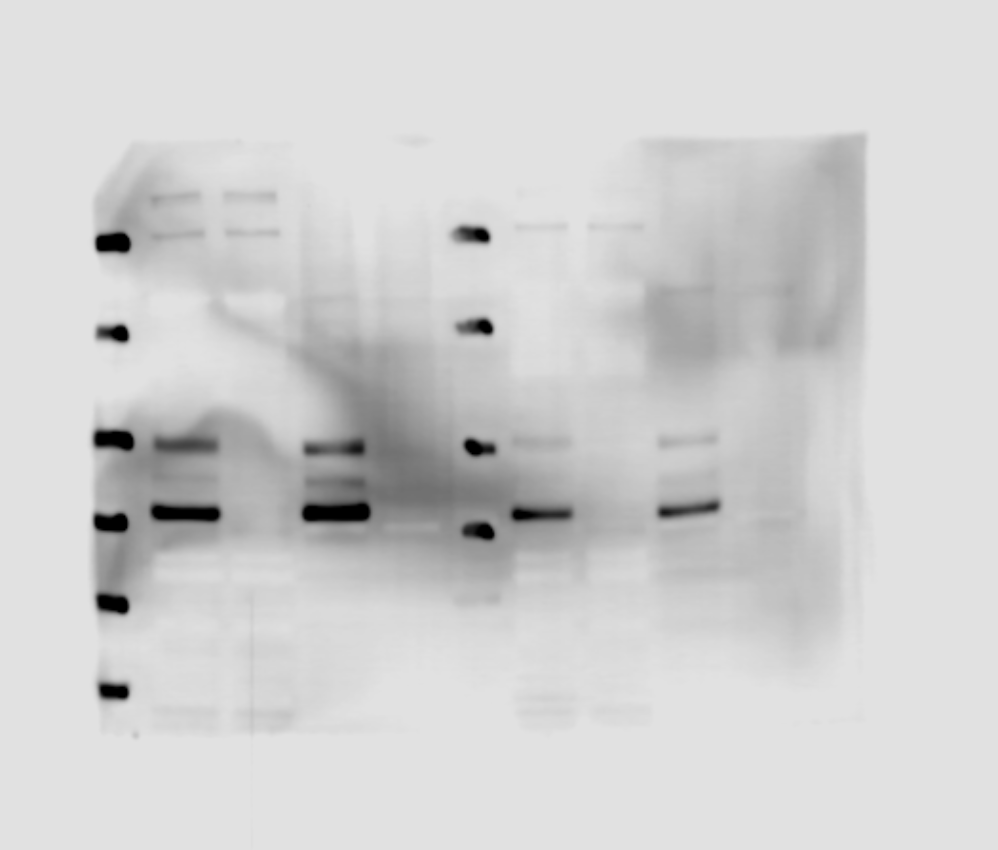

Supplement: Figure 5—source data 2. [file elife-100797-fig5-data2.zip › Figure 5 - Source Data 2/GATA6-IP GATA6 blot Day2.png]

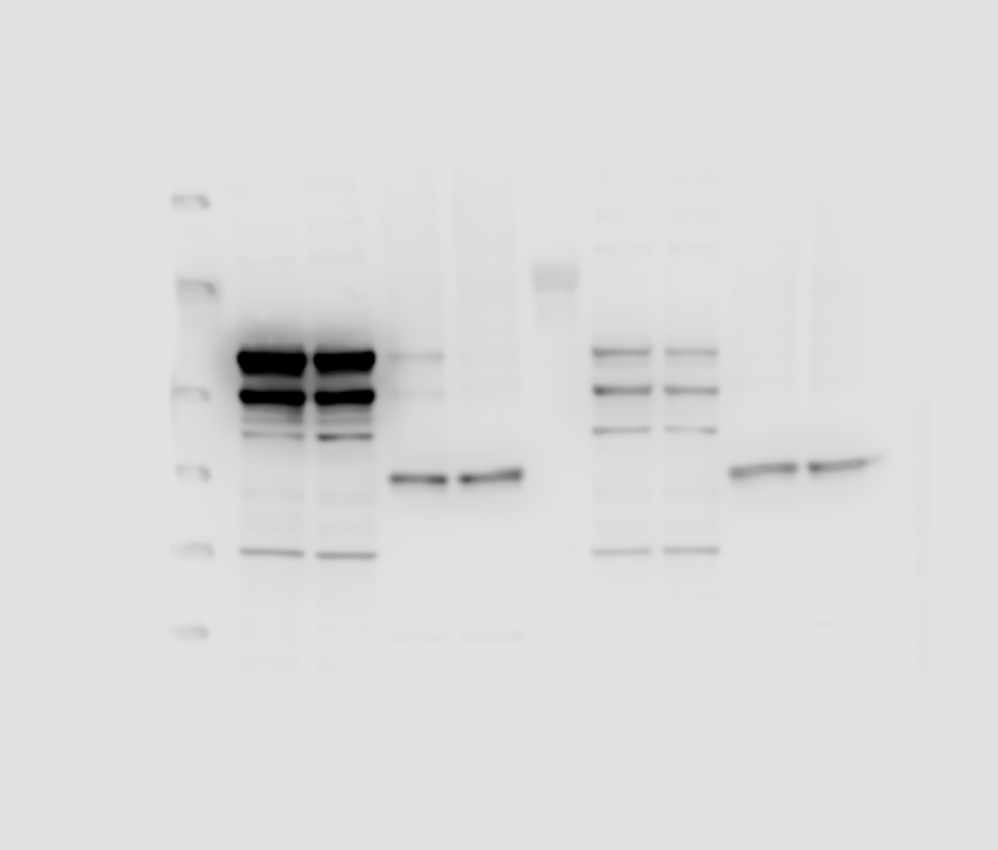

Supplement: Figure 5—source data 2. [file elife-100797-fig5-data2.zip › Figure 5 - Source Data 2/GATA6-IP EOMES blot Day2.png]

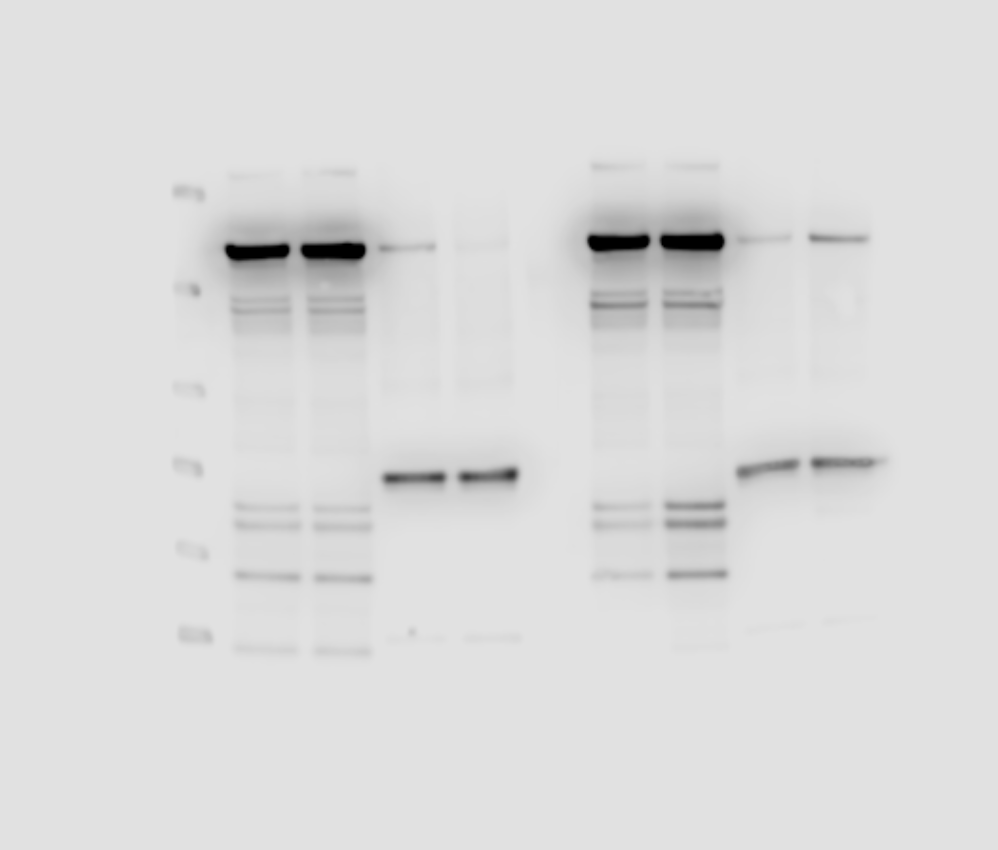

Supplement: Figure 5—source data 2. [file elife-100797-fig5-data2.zip › Figure 5 - Source Data 2/GATA6-IP SMARCC1 blot Day2.png]
