# Supplementary material for: GATA6 regulates WNT and BMP programs to pattern precardiac mesoderm during the earliest stages of human cardiogenesis
Source: eLife. 2025 Mar 13;13:RP100797. doi: 10.7554/eLife.100797 (PMC11906159; doi:10.7554/eLife.100797)
Supplement: Figure 2—source data 1. [file elife-100797-fig2-data1.pdf]

Figure 2 – Source Data 1

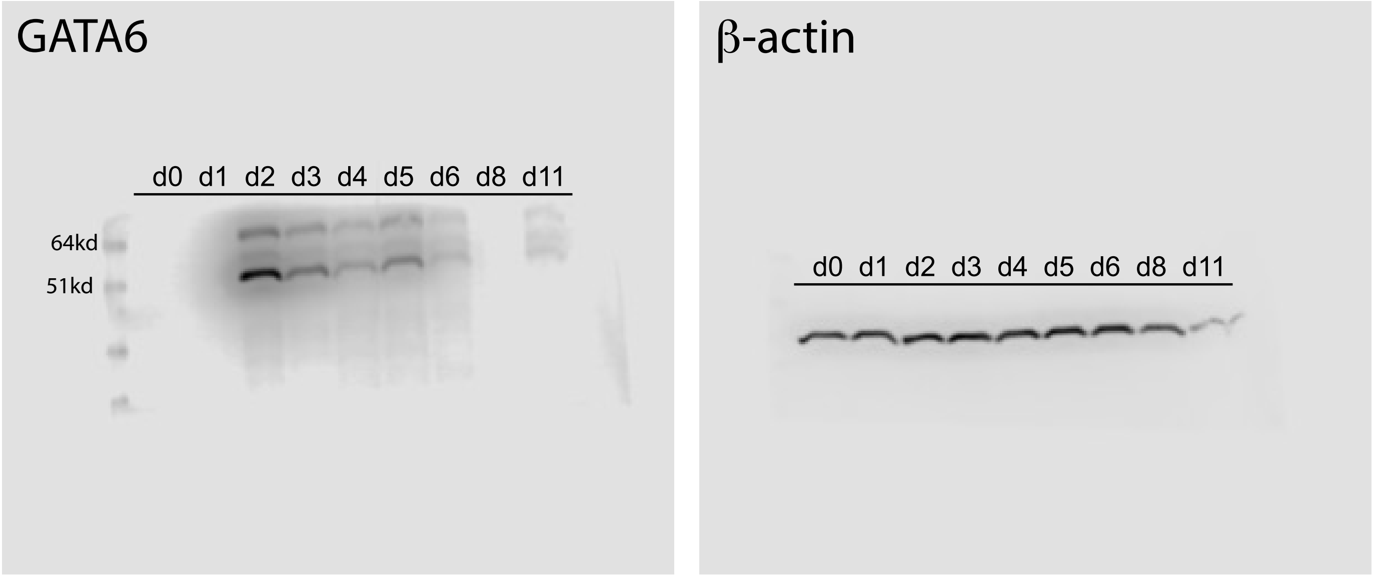

Figure 2 – Source Data 1. Original western blot images corresponding to Figure 2A. Labels indicate the antibody used (top left corners), day of cardiac differentiation, and/or the molecular weight of the relevant ladder bands.
