# Supplementary material for: GATA6 regulates WNT and BMP programs to pattern precardiac mesoderm during the earliest stages of human cardiogenesis
Source: eLife. 2025 Mar 13;13:RP100797. doi: 10.7554/eLife.100797 (PMC11906159; doi:10.7554/eLife.100797)
Supplement: Figure 3—source data 1. [file elife-100797-fig3-data1.pdf]

Figure 3 – Source Data 1

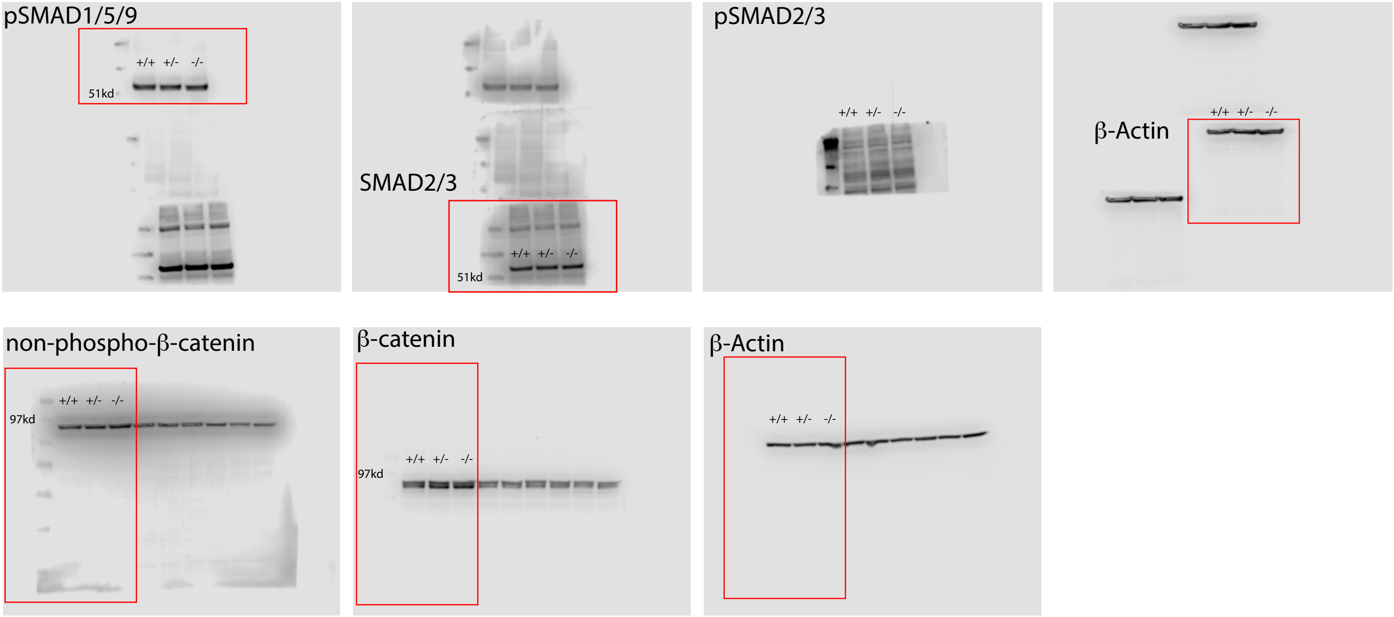

Figure 3 – Source Data 1. Original western blot images labeled corresponding to Figure 3F. The top row of images includes the western blots relating to day 2 +/+, +/-, and -/- samples probed for pSMAD1/5/9, SMAD2/3, and pSMAD2/3 with β-actin as a loading control for this set of experiments. The bottom row of images indicates the western blots relating to day 2 +/+, +/-, and -/- samples probed for non-phospho-β-catenin and β-catenin with β-actin as a loading control for this set of experiments. The red boxes indicate the relevant blots (top row) and sample bands (bottom row) corresponding to Figure 3F.
