# Supplementary material for: GATA6 regulates WNT and BMP programs to pattern precardiac mesoderm during the earliest stages of human cardiogenesis
Source: eLife. 2025 Mar 13;13:RP100797. doi: 10.7554/eLife.100797 (PMC11906159; doi:10.7554/eLife.100797)
Supplement: Figure 5—source data 1. [file elife-100797-fig5-data1.pdf]

Figure 5 – Source Data 1

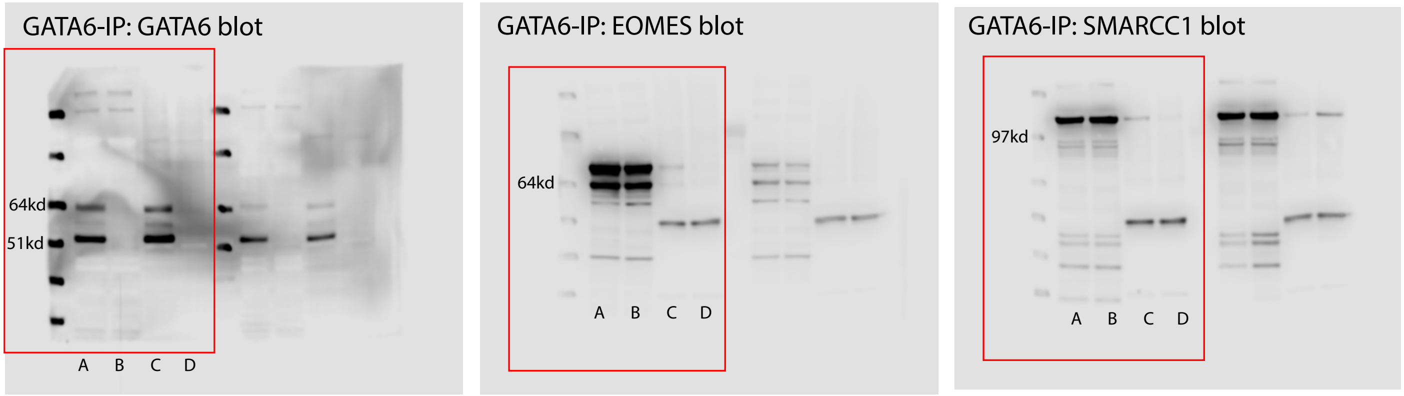

Figure 5 – Source Data 1. Original western blot images corresponding to Figure 5E. Text labels in the top left indicate the antibody used in the GATA6-IP experiment. Red boxes indicate relevant sample bands. A-D indicates individual samples defined as - A: input +/+, B: input -/-, C: GATA6-IP +/+, D: GATA6-IP -/-.
