# Supplementary material for: GATA6 regulates WNT and BMP programs to pattern precardiac mesoderm during the earliest stages of human cardiogenesis
Source: eLife. 2025 Mar 13;13:RP100797. doi: 10.7554/eLife.100797 (PMC11906159; doi:10.7554/eLife.100797)
Supplement: Supplementary file 1. [file elife-100797-supp1.docx]

**Supplementary File 1: GATA6-RIME Enriched Proteins**
