# Supplementary material for: GATA6 regulates WNT and BMP programs to pattern precardiac mesoderm during the earliest stages of human cardiogenesis
Source: eLife. 2025 Mar 13;13:RP100797. doi: 10.7554/eLife.100797 (PMC11906159; doi:10.7554/eLife.100797)
Supplement: Supplementary file 2. [file elife-100797-supp2.docx]

Supplementary File 2: Cell lines, primer sequences, and antibodies used.

**Cell Lines**

| **Name** | **Source** | **Notes** |
| --- | --- | --- |
| H1-GATA6-hESCs | Parent line: WiCell Research Institute, H1 (WA01), NIHhESC-10-0043 | Male |
| GATA6 c.1071delG iPSCs | Yu et al., 2014 (Ref: 15) | Male |
| 293T | ATCC: human embryonic kidney cells, CRL-11268 |  |
| CF1 Mouse Embryonic Fibroblasts, irradiated | MTI Global Stem: CF-1 MEF 2M IRR |  |

**gRNAs sequences**

| **Name** | **Sequence 5' to 3'** | **Cell line used** | **Notes** |
| --- | --- | --- | --- |
| G6-Cr1 | GGCGTTTCTGCGCCATAAGG | H1-GATA6-hESCs |  |
| G6-Cr2 | TTATGGCGCAGAAACGCCG | H1-GATA6-hESCs |  |
| gRNA 1 - F | caccgCACAGCCTGCAGAGCCGCGC | GATA6 c.1071delG iPSCs | gRNA target sequence capitalized |
| gRNA 1 - R | aaacGCGCGGCTCTGCAGGCTGTGc | GATA6 c.1071delG iPSCs | gRNA target sequence capitalized |
| gRNA 2 - F | caccgGTGCAGCACGGGGTCTCGAA | GATA6 c.1071delG iPSCs | gRNA target sequence capitalized |
| gRNA 2 - R | aaacTTCGAGACCCCGTGCTGCAc | GATA6 c.1071delG iPSCs | gRNA target sequence capitalized |

**ssDNA HDR template**

| **Cell Line used** | GATA6 c.1071delG iPSCs |
| --- | --- |
| **Sequence 5' to 3'** | GGCGCCACTGACGCCTGCCTGGCCCGCCGGACCCTTCGAGACCCCGGTGCTGCACAGCCTACAGAGCCGCGCCGGAGCCCCGCTCCCGGTG |

**Primer sequences**

| **Name** | **Sequence 5' to 3'** | **Application** |
| --- | --- | --- |
| G6-iPSC-F | GCCCCTACTCGCCCTACG | PCR genotyping |
| G6-iPSC-R | CACCCGGACGAGAAAGTCCT | PCR genotyping |
| LGR5-cloning-F | TTCGTACGTACTTCGGGCACCATGGA | Full Length LGR5 PCR (using day 2 cDNA). Includes BsiWI site. |
| LGR5-cloning-R | GGCAATTGTTAGAGACATGGGACAAATGCC | Full Length LGR5 PCR (using day 2 cDNA). Includes MfeI site. |
| LGR5-seq1 | GAAAGAAATGCTTTGATGGG | LGR5 Sanger sequencing primer 1 |
| LGR5-seq2 | ATGCATTTTCCACTTTGCCA | LGR5 Sanger sequencing primer 2 |
| LGR5-seq3 | CTGGTGGGAGAATGGGGTTG | LGR5 Sanger sequencing primer 3 |
| CS-TRE-F | ATTTTCGGGTTTATTACAGG | Lentiviral vector Sanger sequencing primer |
| CS-TRE-R | GGAAAGGAGCTGACAGGT | Lentiviral vector Sanger sequencing primer |
| HPRT-F | ACCAGTCAACAGGGGACATAA | RT-qPCR |
| HPRT-R | CTTCGTGGGGTCCTTTTCACC | RT-qPCR |
| NKX2.5-F | AGCCGAAAAGAAAGAGCTGTGCG | RT-qPCR |
| NKX2.5-R | GACCTGCGCCTGCGAGAAGAG | RT-qPCR |
| TBX20-F | GGCGACGGAGAACACAATCAA | RT-qPCR |
| TBX20-R | CTGGGCACAGGACGACTTC | RT-qPCR |
| TBX5-F | AGCAGTGACTTCCTACCAGAAC | RT-qPCR |
| TBX5-R | TGACATTCTGTGCAGCTCCAT | RT-qPCR |
| GATA6-F | CTGCGGGCTCTACAGCAAG | RT-qPCR |
| GATA6-R | GTTGGCACAGGACAATCCAAG | RT-qPCR |
| GATA4-F | AAAGAGGGGATCCAAACCAG | RT-qPCR |
| GATA4-R | TTGCTGGAGTTGCTGGAAG | RT-qPCR |
| MEF2C-F | CTGGTGTAACACATCGACCTC | RT-qPCR |
| MEF2C-R | GATTGCCATACCCGTTCCCT | RT-qPCR |
| ALDH1A2-F | GGAGTCCCTTTGACCCCACCACT | RT-qPCR |
| ALDH1A2-R | CCCTTTCGGCCCAGTCCTTTGC | RT-qPCR |
| LGR5-F | GTTTCCCGCAAGACGTAACT | RT-qPCR |
| LGR5-R | CAGCGTCTTCACCTCCTACC | RT-qPCR |
| T-F | ACCCAGTTCATAGCGGTGAC | RT-qPCR |
| T-R | CCATTGGGAGTACCCAGGTT | RT-qPCR |
| EOMES-F | CTGCCCACTACAATGTGTTCG | RT-qPCR |
| EOMES-R | GCGCCTTTGTTATTGGTGAGTTT | RT-qPCR |
| ISL1-F | GCGGAGTGTAATCAGTATTTGGA | RT-qPCR |
| ISL1-R | GCATTTGATCCCGTACAACCT | RT-qPCR |
| HAND1-F | GTGAGAGCAAGCGGAAAAG | RT-qPCR |
| HAND1-R | GTG CGT CCT TTA ATC CTC TTC | RT-qPCR |
| SMARCD3-F | TCAATGGGGACAAGTATTTCCAG | RT-qPCR |
| SMARCD3-R | GTTGGCCGTGGATAGGAGG | RT-qPCR |

**Antibodies**

| **Antibody** | **Source** | **Application** |
| --- | --- | --- |
| mouse monoclonal α-Actinin | Sigma-Aldrich A7811 | immunocytochemistry 1:200 |
| rabbit polyclonal NANOG | Cell Signaling Technology 3850 | immunocytochemistry 1:250 |
| rabbit polyclonal SOX2 | Invitrogen 48-1400 | immunocytochemistry 1:100 |
| mouse monoclonal cardiac troponin T | Invitrogen MA5-12960 | immunocytochemistry 1:200, flow cytometry 1:500 |
| rabbit monoclonal GATA6 | Cell Signaling Technology 5851 | western blotting 1:1000 |
| mouse monoclonal !3-actin | Sigma-Aldrich A1978 | western blotting 1:50000 |
| mouse monoclonal anti-human KDR PE conjugated | R&D Systems FAB357P-100 | flow cytometry 10µL per million cells |
| mouse monoclonal anti-human PDGFRa PE conjugated | R&D Systems FAB1264A | flow cytometry 10µL per million cells |
| goat polyclonal anti-human/mouse Brachyury PE-conjugated | R&D Systems IC2085P | flow cytometry 1:40 |
| rabbit polyclonal SMAD2/3 | Cell Signaling Technology 3102 | western blotting 1:500 |
| rabbit monoclonal Phospho-SMAD2 (Ser465/467)/SMAD3 (Ser423/425) (D27F4) | Cell Signaling Technology 8828 | western blotting 1:500 |
| rabbit monoclonal Phospho-SMAD1 (Ser463/465)/ SMAD5 (Ser463/465)/ SMAD9 (Ser465/467) (D5B10) | Cell Signaling Technology 13820 | western blotting 1:500 |
| rabbit monoclonal non-phospho (Active) β- Catenin (Ser33/37/Thr41) (D13A1) | Cell Signaling Technology 8814 | western blotting 1:1000 |
| rabbit polyclonal β-Catenin | Cell Signaling Technology 9562 | western blotting 1:1000 |
| rabbit monoclonal SMARCC1/BAF155 (D7F8S) | Cell Signaling Technology 11956 | western blotting 1:1000 |
| rabbit polyclonal TBR2 / Eomes | Abcam ab23345 | western blotting 1:500 |
| goat anti-mouse IgG1 Alexa Fluor™ 488 | Invitrogen A-21121 | immunocytochemistry 1:500 |
| goat anti-rabbit Alexa Fluor™ 488 | Invitrogen A-11008 | immunocytochemistry 1:1000 |
| goat anti-mouse Alexa Fluor™ 488 | Invitrogen A-10667 | immunocytochemistry 1:500 |
| goat anti-mouse Alexa Fluor™ 647 | Invitrogen A-21236 | flow cytometry 1:400 |
| goat anti-rabbit HRP | Bio-Rad 1706515 | western blotting 1:2000 - 1:10000 |
| goat anti-mouse HRP | Bio-Rad 1706516 | western blotting 1:2000 - 1:10000 |
| mouse monoclonal IgG1 APC-conjugated | R&D Systems IC002A | flow cytometry 10µL per million cells |
| mouse monoclonal IgG1 PE-conjugated | R&D Systems IC002P | flow cytometry 10µL per million cells |
| goat polyclonal IgG PE-conjugated | R&D Systems IC108P | flow cytometry 1:40 |
